# Improving the Diagnostic Workup of Vertigo: A Multidisciplinary Review

**DOI:** 10.51894/001c.158361

**Published:** 2026-03-04

**Authors:** Vivek Patil, Suhas B. Nagappala, Ananya Murali, Kristof Pusztai, Jithin John, Lauren Ghannam, Abigail Entz, Andrew Ross

**Affiliations:** 1 College of Medicine - Tucson University of Arizona, , Tucson, AZ, USA; 2 School of Medicine Wayne State University, Detroit, MI, USA; 3 Statistics Columbia University, New York, NY, USA; 4 Otolaryngology-Head and Neck Surgery Detroit Medical Center, Detroit, MI, USA; 5 Henry Ford Hospital, Detroit, MI, USA; 6 College of Osteopathic Medicine Michigan State University, Detroit, MI, USA

**Keywords:** vertigo, nystagmus, algorithms, artificial intelligence, machine learning, Dix-Hallpike

## Abstract

This Clinical (Narrative) Review addresses potential improvements to the diagnostic workup of vertigo. Despite substantial healthcare expenditures, diagnostic accuracy for vertigo remains suboptimal, and many patients undergo extensive testing without timely identification of the underlying cause. The available literature, with a focus on articles published after 2015, was narratively reviewed for novel risk stratification metrics, clinical pathways, questionnaires, and technological tools that have been developed and studied for improving the diagnostic efficiency and accuracy of vertigo. Recent developments in the workup of vertigo are diverse and show potential for accurately stratifying patients by peripheral versus central etiology. As with any new clinical tools, there are barriers when it comes to implementing them into an emergency or primary care setting. AI/ML-based models are promising; however, further study is needed. These tools may support improved efficiency of the evaluation and management of patients who present with vertigo.

## INTRODUCTION

This Clinical (Narrative) Review centers on vertigo, a common chief complaint in primary care and emergency department (ED) settings, described by patients as a sensation of spinning and whirling. This is in contrast to dizziness, which is typically described as lightheadedness or a feeling of unsteadiness as if one is about to lose consciousness. The typical first step is to discern whether a patient is experiencing central or peripheral vertigo. Central vertigo is defined as vertigo attributed to pathology within the central nervous system, including strokes affecting the brainstem, vertebrobasilar ischemia, vestibular migraine, cerebellopontine angle tumors, meningitis, and multiple sclerosis.^1^ Peripheral vertigo, which accounts for approximately 70% of vertigo cases, arises from dysfunction in the peripheral vestibular system.^2^ This includes diseases such as benign paroxysmal positional vertigo (BPPV), vestibular neuritis, and Ménière disease. BPPV, which comprises over one-third of all vertigo cases, is caused by dislodged calcium carbonate crystals, also called canaliths, in the inner ear.^2^ Vestibular neuritis is a viral inflammatory disorder affecting the eighth cranial nerve, while Ménière disease occurs due to endolymphatic hydrops.^3^

Assessing the risk of central etiology can include a review of vitals, neurological and cardiac evaluation, and imaging.^1^ Central etiology is of immediate concern if a patient presents with additional focal neurologic symptoms, such as speech difficulty, diplopia, hearing loss, ataxia, or dysmetria; in addition to comorbidities such as poorly controlled hypertension, hyperlipidemia, prior stroke, or prothrombotic conditions. The TiTrATE approach focuses on the timing of symptoms, triggers, and targeted examination to explore the cause of vertigo. If vertigo occurs with positional changes, BPPV is a probable cause. The gold standard maneuver to test for BPPV is the Dix-Hallpike maneuver (DHM), which entails turning the patient’s head 45° to one side while seated upright and rapidly reclining them into a supine position. A positive DHM is declared if the patient experiences vertigo and characteristic positional nystagmus, specifically torsional or up beating nystagmus for posterior canal BPPV, following the maneuver. If there is no known trigger for the vertigo, the underlying issue might be Ménière’s disease or a primary psychiatric issue. Lastly, if vertigo is continuous, it points to vestibular neuritis or central vertigo.^4^

Another way to differentiate peripheral and central vertigo is using the HINTS approach which evaluates head-impulse saccades, the direction of a nystagmus, and gaze dedication with the cover-uncover test; a peripheral etiology is supported by the presence of a saccade, horizontal nystagmus, and a lack of gaze deviation, while a central etiology is supported by a lack of saccade, vertical or rotational nystagmus, and a vertical gaze deviation.^5^

Given its broad differential diagnosis, the work-up for vertigo often involves MRI, CT, and physical examinations performed by physicians across multiple specialties to rule out central causes, often incurring substantial costs.^6^ Studies suggest that neuroimaging for vertigo and dizziness is overused.^7^ Meanwhile, low-cost physical exam maneuvers such as HINTS are underutilized.^8^ This emphasizes the need for improved diagnostic efficiency and optimized management. The literature reveals a vast array of interventions aimed at improving vertigo diagnostics, spanning imaging optimization, education, patient questionnaires, clinical decision support, and novel artificial intelligence tools. These collectively address challenges in the diagnostic pathway from initial triage to treatment. From this Clinical (Narrative) Review, clinicians will understand the latest diagnostic advances in vertigo over the last decade. They will also be able to discuss the emerging tools to help differentiate a peripheral etiology versus central etiology for vertigo.

### Literature Identification

Literature was selected based on clinical relevance, recency, and impact, in a non-systematic manner.

## SUMMARY OF EVIDENCE

### Provider Educational Sessions

Educational sessions and materials for providers have emerged as a strategy for improving vertigo management. Robust evidence comes from Kerber et al.’s multimodal intervention, combining hands-on BPPV education for emergency department (ED) providers with distribution of digital and print diagnostic aids. This increased documented use of the DHM and canalith repositioning maneuver (CRM) while reducing head CT orders, without impacting 90-day stroke risk.^9^ Similarly, Kozlowski et al.’s intervention, involving joint educational rounds (by the ED, diagnostic imaging program, and stroke neurology teams) and distribution of a diagnostic aid (a mnemonic-based tool), produced a 38% relative reduction in CTA ordering (with especially strong effects for vertigo presentations), without compromising essential CTA usage for stroke detection.^10^

However, the relationship between education and neuroimaging use may be context-dependent. Despite including education on diagnostic tests and treatments such as Dix-Hallpike, supine roll, and CRM alongside a standardized clinical algorithm, Arbab et al.’s intervention did not significantly decrease CT scan usage, though it did counteract the declining trend in BPPV diagnostic and treatment practices. This divergence from Kozlowski et al.’s findings might be because additional medicolegal, institutional, and clinical variables likely modulate educational interventions’ impact on imaging behaviors.^10,11^

Novel educational delivery methods show promise for enhancing clinical skills. Ursat et al. explored HINTS training for emergency physicians using a mannequin-based virtual reality simulator, finding that physicians receiving hands-on simulator training achieved substantially higher HINTS diagnostic performance than lecture-only controls, with sustained performance improvement at 6 months.^12^ However, the failure to improve physician self-confidence despite these objective performance improvements indicates a need for evaluating optimal training frequency. Moreover, performance on a simulator cannot be directly applied to real-world scenarios, and examiner training can be difficult to standardize. Complementary approaches include Meurer et al.’s randomized controlled vignette-based program, which markedly increased planned DHM and CRM usage, and Omron et al.’s multimodal intervention combining online gaming, lectures, and hands-on training, which boosted the proportion of ED residents comfortable discharging vestibular neuritis patients without CT from 68% to 96%.^13,14^ The evidence from these multimodal educational programs suggests that interactive training is more impactful than pure didactics.

### Abbreviated Imaging

Abbreviated MRI protocols, focused MRI protocols often less than 10 minutes that rely on thin-section axial and coronal diffusion-weighted imaging, may offer a promising middle ground between diagnostic accuracy and resource efficiency.^15–17^ In Shareef et al.’s ED study, abbreviated MRI detected acute intracranial pathology with greater sensitivity than CT/CTA.^17^ The practical implications were identified by Tu et al.’s study of 14,204 patients, where abbreviated MRI protocols resulted in significantly shorter turnaround times and length of stays compared to standard MRI.^16^ This added to earlier findings that patients with vertigo who underwent abbreviated MRI had improved results compared to CTA alone regarding critical result identification and secondary stroke prevention medication changes.^15^

This evidence positions abbreviated MRI as an imaging alternative that may improve speed of care over standard MRI and could improve outcomes compared to CT/CTA. Considering that abbreviated MRI can be shortened to 5 minutes, and recent clinical practice guidelines recommend MRI over CT, further exploring the utility of abbreviated MRI is worthwhile.^17,18^ Of course, efficiency should not come at the expense of patient safety; traditional imaging approaches continue to be reliable for central cause exclusion, and should be applied when clinically logical.

### Standard Patient Questionnaires

Patient questionnaires are tools that allow simple and accessible screening of patients complaining of vertigo. Muraleedharan et al. designed a five-question BPPV instrument with Yes/No and Right/Left answers, asking about dizziness duration and description, triggering movements, and symptom patterns between attacks. It achieved 94.5% sensitivity and 91.22% specificity for BPPV diagnosis, with 93.24% accuracy when excluding siding.^19^ This performance suggests high utility for triage in primary care settings where specialist testing might not be available, potentially facilitating appropriate referrals. Attempts to expand diagnostic scope have seen modest success, shown by Suvanich et al.’s broader dizziness questionnaire. It included 20 questions (about time course, triggering factors, and symptoms) with Yes/No answers, arranged algorithmically to differentiate among vestibular and non-vestibular disorders. The questionnaire showed moderate agreement with experienced otoneurologists, with BPPV and VM diagnosis having the highest concordance.

This evidence suggests these tools cannot substitute for formal vestibular function tests for definitive diagnosis, but may offer valuable screening utility in resource-limited settings where specialist evaluation is delayed or unavailable.^20^ Future questionnaires should emphasize asking about factors positively and negatively associated with stroke risk.^21^

### Physical Examination Modifications and Additional Diagnostic Tests

Efforts to enhance or supplement traditional bedside assessment have yielded mixed results. Godha et al. found cervical VEMP testing demonstrated 83.3% specificity but just 34.2% sensitivity for BPPV, indicating limited screening utility, but potential value for ruling in BPPV.^22^ More pertinent for practical implementation, Jeon et al. found equivalent diagnostic values between standard and modified (“pillow-under-shoulders”) Dix-Hallpike maneuvers. Importantly, most patients felt less inconvenienced during the modified test.^23^ Especially promising for optimizing flow is an abbreviated Dix-Hallpike maneuver, which, without requiring an examination chair/table, achieved 80% sensitivity and 95% specificity for posterior BPPV diagnosis.^24^ Several avenues for modified bedside assessments for improved patient and provider experiences exist, but viability in replacing traditional assessments may be limited.

### Risk Stratification Measurements

A plethora of novel risk metrics for central etiology have been explored, potentially offering clinicians evidence-based tools to guide imaging decisions. In multicenter evaluation, the STANDING algorithm demonstrated 88.2% sensitivity and 91.6% specificity for central pathology, and was associated with reduced non-contrast head CT usage and length of stay compared to usual care.^25^ The TiTrATE-STANDING Adapted algorithm (combining STANDING, HINTS, and TiTrATE) revealed 90% sensitivity and 57.9% specificity. Such integrated algorithms may streamline diagnostics, but improvements to ensure the combined model performs equal to or better than its parts are necessary.^26^ Menekse et al.’s evaluation of TriAGe+ scores for predicting acute ischemic stroke in ED patients with vertigo/dizziness revealed 0.979 AUC, achieving higher predictive accuracy than ABCD scores, especially in cases without sensory loss or focal weakness.^27^ Notably, the ABCD-3I score (requiring advanced imaging) achieved predictive accuracy similar to TriAGe+, underscoring the value of the easily determined TriAGe+.

The Sudbury Vertigo Risk Score incorporates stroke risk factors (male, age >65, diabetes and/or hypertension) and additional neurologic deficits (motor/sensory and/or cerebellar), with BPPV diagnosis being protective against serious causes of vertigo. It achieved 100% sensitivity for predicting stroke across both derivation and multicenter testing with 4559 cases, with specificity around 70%.^28,29^ However, concerns emerged that BPPV misdiagnosis could lead to overlooking central causes, and that low MRI utilization in the study (56/2078 patients) potentially missed stroke diagnoses.^30^ The lead author, Ohle, responded that BPPV misdiagnosis risk was managed by emergency physician training, and that low MRI utilization was compensated for with chart reviews, phone interviews, and committee evaluations.^31^ Further large-sample testing would be worthwhile to verify diagnostic utility.

The EMERALD Vertigo Rule, developed with recursive partitioning analysis, incorporates blood pressure, blood sugar, LDH level, WBC count, sex, and the presence of vomiting and headache or neck pain. In multicenter testing, it achieved 100% sensitivity and 20.0% specificity, indicating strong potential for ruling out central etiology.^32^ Given the seriousness of central etiology, approaching 100% sensitivity with these measures is crucial, although multiple metrics can be combined in clinical practice. The Sudbury Vertigo Risk Score could be further enhanced by accounting for the severity of hypertension and diabetes as the EMERALD Vertigo rule does or incorporating additional risk factors such as atrial fibrillation, autoimmune conditions, or sickle cell disease. Current risk metrics show promise in enhancing decision-making, as suggested by the evidence. However, their cost-effectiveness remains to be seen, as false positives due to subpar specificities may impede neuroimaging reduction.

### Non-AI/ML Novel Management Algorithms and Clinical Pathways

Standardized management algorithms show strong potential for improving accuracy and efficiency. Filipopulos et al.’s novel three-level PoiSe algorithm, intended for future deployment in primary care and ED settings, was tested at the University of Munich’s affiliated hospital. Using medical history items including neurologic deficits, accompanying headache and ear symptoms or nystagmus, and simple clinical examinations, it achieved 71% overall accuracy, 93.8% sensitivity for cerebrovascular events, and specificities of >95.2% for non-vascular vestibular diagnoses.^33^ Practical implications were illustrated by Sandlund et al., whose novel management algorithm for dizzy patients was tested at the Umeå University Hospital. The algorithm doubled Dix-Hallpike usage and BPPV diagnosis rates, while simultaneously reducing hospital stay, neuroradiological testing, and per-patient costs ($2,561 to $1,808), without increasing adverse events.^34^ This demonstrates that systematic approaches can achieve the dual goals of better diagnostics and lower resource utilization. Machner et al., also proposed a novel clinical pathway based on physical exam findings to determine whether to order neuroimaging in acutely dizzy patients.^35^ However, in ED and primary care settings, implementation of these pathways may be limited by the need for department-wide provider education and the time needed for performing physical exams.

### Novel Management Algorithms (ML/AI)

Artificial intelligence algorithms in vertigo diagnosis are increasingly numerous and diverse, using varied approaches, targets, and technologies. Many of these emerging tools show proficiency at their respective tasks in testing environments. However, for practical efficiency improvements, physicians must trust AI recommendations more than the medicolegal assurance of ordering scans. Based on the reviewed evidence, we recommend further investigation into AI tool implementation and ethics before extensive clinical deployment.

### Disease Specific Models

Focused algorithms for specific diseases are promising. Han et al. created a predictive model for BPPV based on eight key clinical variables determined via LASSO regression and random forest analysis, achieving high diagnostic accuracy and identifying metabolic markers potentially related to BPPV pathogenesis.^36^ Tang et al. designed an algorithm using XGBoost, a scalable tree-boosting machine learning model, that effectively distinguished BPPV from non-BPPV, with a strong ability to further classify non-BPPV patients with Meniere’s disease, sudden sensorineural hearing loss, and vestibular migraine.^37,38^ Soylemez et al. explored translating such models into accessible tools, using medical histories and vertigo clinical features to train and test ML models for posterior canal BPPV, finding that random forest performed the best. They subsequently developed a mobile application, potentially allowing easy self-assessment for patients with dizziness.^39^ Based on these findings, we believe investigation into whether such self-screening might reduce unnecessary ED visits is warranted.

For acute vestibular syndrome, Korda et al.’s neural network, trained and tested on minimally processed video head impulse data, performed equivalently to traditional vestibulo-ocular reflex gain classification.^40^ This equivalence indicates potential for AI to replicate expert interpretation, which may increase diagnostic capabilities in ED and primary care settings.

### Broader Classification

Models incorporating diverse data types have achieved impressive testing accuracy across multiple diagnostic categories. Zhao et al.’s model, based on 32 medical history items and 9 physical signs, achieved 98.11% accuracy across 16 dizziness/vertigo diseases in 1,003 cases. Notably, it demonstrated superior robustness to physical sign perturbations compared to baseline models, an essential consideration for real-world utility where examination quality may vary.^41^ Callejas et al.’s CatBoost model illustrated the importance of combining clinical expertise with algorithmic features, performing markedly better when using clinician-guided features.^42^ This evidence indicates that AI models benefit substantially from informed feature selection rather than purely data-driven approaches.

### Computer Vision Nystagmus Analysis

Computer vision applications for nystagmus detection represent a rapidly evolving subfield with major clinical implications. One 2D-convolutional model, based on review of over 90,000 video clips, achieved strong performance for horizontal and vertical nystagmus, with good performance for predicting the BPPV-affected canal. However, torsional component detection lagged, indicating a torsional nystagmus training gap.^43^ The Torsion-aware Bi-stream Identification Network was created to address this gap, achieving 85.73% accuracy during frame-level identification testing, with robustness in correcting for eyelid or eyelash occlusion.^44^ Li and Yang’s BiLSTM model, examining past and future video frames for torsional nystagmus, improved recognition accuracy over previous models.^45^ Mun et al. then developed a model with higher sensitivity and F1-score than predecessors.^46^

These consecutive successes in torsional nystagmus identification are significant, given the difficulty of observing torsional motion and its high yield in posterior canal BPPV.^44^ Based on its ability to make subtle findings accessible, we believe this technology has considerable potential to benefit clinicians in the near future.

The portability and efficiency of such models (which, in the past, have often required significant processing power) is an important consideration. One lightweight model, capable of achieving high frame rates even on consumer-grade workstations, reached correlation coefficients (against ground truth) of >0.99 for real-time horizontal and vertical nystagmus detection in posterior canal BPPV videos.^47^ An exceptionally portable model is Bastani et al.’s EyePhone, which utilizes the iPhone 13 ProMax’s FACE ID front camera. When considering videooculography data as the true positive, EyePhone achieved high predictive power (AUC 0.87) for both horizontal and vertical nystagmus detection.^48^ These portable solutions could extend diagnostic capabilities to under-resourced primary care and ED settings where specialized equipment is unavailable. Additionally, aEYE, a deep learning model tested on recordings from the AVERT clinical trial, showed minimal decline in nystagmus detection ability with decreasing image resolution, indicating viability in settings with limited video capturing ability.^49^

### Large Language Models

General-purpose large language models (LLMs) have shown diagnostic capabilities, but significant performance gaps persist. Testing of the open-source LLaMa-3.1-8B revealed performance falling behind earlier ML-based models. The model’s moderate level of agreement with otoneurologists was comparable to agreement between ED physicians and otolaryngologists, meaning it approximates non-specialist performance.^50^ ChatGPT-4o performed similarly to one-year-experience otologists but inferiorly to five-year-experience otologists, with strong performance for BPPV and schwannoma but weakness for vestibular migraine and sudden sensorineural hearing loss.^51^ These results indicate that considerable refinement of LLMs is needed to achieve expert-level diagnostic capabilities, though their current performance may offer modest value in settings lacking specialist access.

## DISCUSSION

The existing literature offers considerable tools and strategies for enhancing the diagnostic workup of vertigo that could be implemented in hospital and outpatient settings.

Modified bedside examinations can improve accessibility and may be optimal in niche scenarios, but are unlikely to replace traditional assessments. Abbreviated imaging methods show potential for reducing turnaround times, provided that traditional imaging is still used when clinically recommended and necessary. Clinical decision rules provide support during decision-making, though further testing in diverse clinical settings is necessary. Providers’ clinical judgment is critical during implementation of all these tools.

Education for ED physicians about the DHM and CRM included a flexible educational session taught by other physicians, video aids, and continued refresher courses. Physical exam modifications such as the pillows-under-shoulders DHM and abbreviated DHM can be taught as well, as they can be useful when available equipment or patient discomfort limits a traditional DHM.^23,24^ A pillows-under-shoulders version of the Epley maneuver has also shown comparable therapeutic efficacy to a standard Epley.^52^

Many patients experiencing vertigo undergo CT or MRI; this raises the question of whether neuroimaging is relied upon too heavily by healthcare providers.^53^ While it may seem beneficial with minimal downsides, neuroimaging introduces a significant amount of time and costs, and should only be done if there is a notable benefit to management.^54^ However, it is extremely important to identify vascular pathology, such as a stroke or vertebral artery dissection. Abbreviated MRI protocols may easily be implemented as they minimally impact the ED workflow and can save a significant amount of time; however, it is worth noting that routine MRIs may be inaccurate for small infarcts within 48 hours.^7^ With refinement, risk stratification metrics such as the Sudbury vertigo risk score, Menekse et al.’s TriAGe+ score, and the EMERALD vertigo rule, can be employed to dictate the need for neuroimaging.^16,28^

Patient questionnaires may serve a similar role to risk stratification scores; in conjunction with a review of comorbidities and vitals, they may also be useful in determining need for neuroimaging. Meanwhile, widespread application of AI/ML models in vertigo diagnostics remains in its infancy, and integration into clinical settings requires rigorous evaluation and phased implementation. Zhao et al.’s broad diagnostic model has shown impressive accuracy compared to relying on physical exam findings alone, raising the question of whether the potential diagnostic improvements justify the time and costs required to implement AI models.^41^ While computer vision nystagmus analysis is promising, the models seem to require further adjustment before implementation in clinical settings. Bastani et al.’s EyePhone is particularly promising, as it is integrated within the iPhone 13’s Face ID feature, making it attainable and uncomplicated for many providers.^48^

LLMs may be useful for initial triage of patients and determining the need for neuroimaging, but require further adjustments for establishing specific diagnoses. However, the advent of AI tools in healthcare has raised concerns about patient data privacy, so implementation may involve substantial legal hurdles to health systems.^55^ Training data bias and generalizability of results beyond testing datasets are additional concerns. AI/ML models often demonstrate impressive performance on the specific task they were trained for, but there is currently no model that performs all necessary tasks for vertigo workup. Deciding which interventions and tools to pursue may be institution-specific, but should involve weighing the effort needed for implementation with the potential impact in improving efficiency and patient costs and length of stay. Ultimately, future deployment of novel AI/ML models requires substantial model refinement, real-world testing, and consideration of ethical and practical concerns.

### Limitations

The narrative design of this article may limit the strength of findings. Literature was non-systematically selected based on recency, clinical relevance, and impact, meaning the article may not encompass all novel improvements to the workup of vertigo. The heterogeneity of selected literature may also reduce the rigor of investigation into each category of intervention. Publication bias may also be present.

## CONCLUSIONS

There is an emerging array of tools and interventions that providers may consider to improve diagnostic accuracy and reduce times and costs in working up patients presenting with vertigo. These tools can support clinical judgement as well as multidisciplinary collaboration between physicians. While the application of AI and ML tools is in its early stages, considerable potential exists for improving efficiency by utilizing risk stratification metrics, non-AI/ML management algorithms, and questionnaires to help inform decisions regarding neuroimaging. Additional approaches such as abbreviated imaging pathways and physical exam maneuvers, as well as educational sessions for providers regarding these tools, warrant further exploration. Future studies are needed to determine whether implementing these improved pathways might reduce medical costs and improve the efficiency of treating patients with vertigo. These studies may then help guide the training of clinicians who encounter vertigo in an emergency or primary care setting.

### Conflict of Interest

None.

### Ethical Approval and Informed Consent

There are no human or animal participants in this study and informed consent is not required.

## Data Availability

Data sharing is not applicable to this article as no datasets were generated or analyzed during the current study.
